# Tumor Vascular Permeability to a Nanoprobe Correlates to Tumor-Specific Expression Levels of Angiogenic Markers

**DOI:** 10.1371/journal.pone.0005843

**Published:** 2009-06-09

**Authors:** Efstathios Karathanasis, Leslie Chan, Lohitash Karumbaiah, Kathleen McNeeley, Carl J. D'Orsi, Ananth V. Annapragada, Ioannis Sechopoulos, Ravi V. Bellamkonda

**Affiliations:** 1 Wallace H. Coulter Department of Biomedical Engineering, Georgia Institute of Technology/Emory University, Atlanta, Georgia, United States of America; 2 Department of Radiology and Winship Cancer Institute, Emory University School of Medicine, Atlanta, Georgia, United States of America; 3 School of Health Information Sciences, University of Texas at Houston, Houston, Texas, United States of America; Health Canada, Canada

## Abstract

**Background:**

Vascular endothelial growth factor (VEGF) receptor-2 is the major mediator of the mitogenic, angiogenic, and vascular hyperpermeability effects of VEGF on breast tumors. Overexpression of VEGF and VEGF receptor-2 is associated with the degree of pathomorphosis of the tumor tissue and unfavorable prognosis. In this study, we demonstrate that non-invasive quantification of the degree of tumor vascular permeability to a nanoprobe correlates with the VEGF and its receptor levels and tumor growth.

**Methodology/Principal Findings:**

We designed an imaging nanoprobe and a methodology to detect the intratumoral deposition of a 100 nm-scale nanoprobe using mammography allowing measurement of the tumor vascular permeability in a rat MAT B III breast tumor model. The tumor vascular permeability varied widely among the animals. Notably, the VEGF and VEGF receptor-2 gene expression of the tumors as measured by qRT-PCR displayed a strong correlation to the imaging-based measurements of vascular permeability to the 100 nm-scale nanoprobe. This is in good agreement with the fact that tumors with high angiogenic activity are expected to have more permeable blood vessels resulting in high intratumoral deposition of a nanoscale agent. In addition, we show that higher intratumoral deposition of the nanoprobe as imaged with mammography correlated to a faster tumor growth rate. This data suggest that vascular permeability scales to the tumor growth and that tumor vascular permeability can be a measure of underlying VEGF and VEGF receptor-2 expression in individual tumors.

**Conclusions/Significance:**

This is the first demonstration, to our knowledge, that quantitative imaging of tumor vascular permeability to a nanoprobe represents a form of a surrogate, functional biomarker of underlying molecular markers of angiogenesis.

## Introduction

Angiogenesis is a critical event enabling tumor growth [1]–[3]. Vascular endothelial growth factor (VEGF) and receptor (VEGFR) signaling pathway plays a pivotal and rate-limiting role in promoting tumor-induced angiogenesis [1], [2]. Angiogenesis correlates not only with the onset of tumor development but also with growth, metastasis and invasion of tumors [4], [5]. It is now established that VEGFR-2 is the major mediator of the mitogenic, angiogenic, and vascular hyperpermeability effects of VEGF [6]–[9].

Like most tumors, breast tumors express many angiogenic factors, such as VEGF and VEGFR, fibroblast growth factor (FGF)-1, FGF-2, angiopoietin-1 and 2, placenta growth factor, hypoxia-inducible factor (HIF)-1α, endothelial cell adhesion molecules (VE-cadherin, PECAM-1), epidermal growth factor, TGF-α and TGF-β [10]–[13]. Notably, various clinical studies demonstrate that higher levels of VEGF overexpression in the tumor correlate with unfavorable prognosis [14]–[16]. Indeed, there is a strong positive correlation between VEGF and VEGFR-2 expression and primary breast cancers and this correlation scales to the degree of pathomorphosis of the primary tissue [17]. Therefore markers of angiogenesis may describe the degree of pathomorphosis of breast cancers and quantitative assessment of these markers may have significant clinical implications. However, it is challenging to non-invasively determine the expression profiles of the angiogenesis-related factors [1]. However, as angiogenesis transiently yields immature vessels, they result in ‘leaky’ microvessels. In the recent past, dynamic contrast-enhanced imaging (DCE-MRI) has been employed to measure tumor vascular permeability to sub 2 nm scale or 5–10 nm scale contrast agents [18]–[20]. Tumor signal enhancement is influenced by the degree of vascularization, vessel permeability, cellularity and interstitial pressure [21]. However, while these approaches are promising, small contrast agents are relatively promiscuous in their leakiness from vessels and diffuse away quickly requiring the use of mathematical models to correlate the dynamic changes in signal enhancement to the physiologic parameters associated with vascular function [22]–[25]. In addition, molecular imaging of angiogenesis biomarkers using positron emission tomography is beginning to be feasible [26], [27].

Here, we investigate a 100 nm nanoprobe, which enables quantification of tumor vessel permeability in a manner that is reliable, and not requiring complex analysis of diffusion dynamics. Due to its size, the 100 nm nanoprobe preferentially accumulates in solid tumors by passive convective transport through leaky endothelium, a phenomenon called the enhanced permeation and retention (EPR) effect [28], [29]. In addition, we investigate whether tumor vascular permeability represents a functional biomarker that scales to the levels of VEGF and VEGFR-2 overexpression in tumors. Traditionally biomarkers are cell surface or intracellular molecular markers – here we suggest that the degree of leakiness of tumor blood vessels ‘integrates’ underlying tumor microenvironmental factors related to angiogenesis, and represents the underlying tumor pathomorphological status. The nanoprobe encapsulates a clinically used iodinated contrast agent for x-ray imaging that enables a quantitative assessment of tumor vessel leakiness using clinically relevant digital mammography [30], [31]. Taking under consideration that mammography is the only method of low cost mass screening of the general population for non-palpable breast cancer [32], is widely available and quick and has a very high spatial resolution, such imaging strategy can be very practical. Using a breast cancer tumor model where the tumor EPR of individual rats varies widely [30], [31], this study evaluates whether this variation of tumor EPR to nanoscale probes, correlates to underlying variation in expression of tumor angiogenic markers.

## Materials and Methods

### Ethics Statement

All animals were handled in strict accordance with good animal practice as defined by the relevant national and/or local animal welfare bodies, and all animal work was approved by the Institutional Animal Care and Use Committee (IACUC) of Georgia Institute of Technology.

### Fabrication of the nanoprobe

The nanoprobe was prepared following previously published methods [31]. A highly concentrated iodine solution (650 mg I/mL) was prepared by dissolving iodixanol powder (lyophilized from Visipaque 320; GE Healthcare, Milwaukee, WI) in ultrapure water under stirring and heating at 70°C. A lipid composition of DPPC, DSPE-PEG_2000_ (Genzyme Pharmaceuticals, Cambridge, MA), and cholesterol (Sigma, St. Louis, MO) in the molar ratio of 55∶5∶40 was used. The lipids were dissolved in ethanol and hydrated with the iodine solution at 70°C followed by sequential extrusion in a Lipex Biomembranes Extruder (Northern Lipids, Vancouver, Canada) to size the liposomes to ∼100 nm. Free, un-encapsulated iodixanol was replaced by a saline solution (300 mM NaCl) with the same osmolarity (596 mOsm/kg water) as the internal iodinated phase of the liposome using a 2-day dialysis with a 100k MWCO dialysis tubing. Following concentration via diafiltration using MicroKros modules (Spectrum Laboratories, Rancho Dominguez, CA) with a 50 nm cutoff pore size, the size of the liposomes was determined by dynamic light scattering (90 Plus Particle Size Analyzer, Brookhaven Instruments, Holtsville, NY). Prior to administration, the final iodine levels were quantified through spectrophotometry (at 245 nm). The liposomal probe contained 75 mg/ml lipids and 165 mg/mL iodine and 100% of the iodine was encapsulated within the liposomes. The average diameter of the probe was 102 nm (standard deviation = 11).

### Mammary adenocarcinoma cell culture

The 13762 MAT B III cells (American Type Culture Collection), a rat mammary adenocarcinoma cell line, were maintained in McCoy's 5A medium supplemented with 10% fetal bovine serum and 1% penicillin-streptomycin under conditions of 5% CO_2_ and 95% humidity at 37°C.

### Animal model

For the tumor model, the 13762 MAT B III cell line was used. Before inoculation, the cells were grown in 90% McCoy's 5A medium and 10% fetal bovine serum. A 0.2 mL aliquot containing 10^6^ cancer cells was subcutaneously injected into the right flank of female Fisher rats with ages of 8–9 weeks (Harlan, Indianapolis, IN). Caliper measurements were used to estimate tumor size and the tumor volume was calculated as: V_tumor_ = (d_1_^2^×d_2_)/2, where d_1_ and d_2_ are the minimum and maximum diameters, respectively.

### X-ray imaging

At day 7 after tumor inoculation (tumor volume ∼500 mm^3^), the animals were imaged using a clinical digital mammography system (Senographe 2000D, GE Healthcare, Milwaukee, WI). To maximize the number of photons with energies above the K-edge of iodine (approx. 33.2 keV) [33], the imaging studies were performed with a 49 kVp, 63 mAs x-ray spectrum, using a rhodium target and a 25 µm thick rhodium filter with an added 0.254 mm thick copper filter.

After the initial imaging session at day 7 after tumor inoculation (t = 0), a group of animals (group A, n = 6) was imaged at defined time points (t = 24, and 72 h) after intravenous (IV) injection of the probe at a dose of 455 mg/kg body weight (b.w.) iodine. Immediately after the last imaging session, the animals were euthanized, tumors were excised, and total RNA was extracted for quantitative real-time reverse transcriptase-polymerase chain reaction (qRT-PCR). Care was taken to obtain the entire tumor without any surrounding tissue.

To evaluate whether the degree of EPR scales to the tumor growth rate, a different group of animals (group B, n = 11) was imaged before (t = 0) and at defined time points (t = 24, 48 and 120 h) after IV injection of the probe at a dose of 455 mg/kg b.w. iodine at day 7 after tumor inoculation. The tumor growth of each animal was monitored at 24 h intervals using caliper measurements. Tumor growth was allowed to progress until the animal showed signs of morbidity, at which point, the animals were euthanized using a CO_2_ chamber.

### Quantitative RT-PCR

qRT-PCR was used to quantify mRNA expression profiles of genes that are closely associated with angiogenesis. Immediately after completion of imaging, the animals of group A were euthanized and tumors were excised. Total RNA was extracted from the entire tumor and muscle (control) of each animal (n = 6) by using RNeasy Maxi RNA extraction kit (Qiagen, Chatsworth, CA) and the protocols therein. Purified RNA was quantified using the Quant-IT Ribogreen reagent (Invitrogen Life Technologies, Carlsbad, CA). Total RNA (2 µg) was converted into cDNA by using Thermoscript RT-PCR system (Invitrogen Life Technologies, Carlsbad, CA). Primers targeting VEGF-A (NM_031836.1) Forward– CGTCTACCAGCGCAGCTATTG and Reverse- CACACAGGACGGCTTGAAGAT; KDR (VEGFR-2) (NM_013062.1) Forward- TTGGCAAATACAACCCTTCAGAT and Reverse-  CACTCAGTCACCAACACCCTTTC; and endogenous control hypoxanthine phosphoribosyltransferase-1 (HPRT1) (NM_012583.2) Forward – TGTTTGTGTCATCAGCGAAAGTG and Reverse - CTGCTAGTTCTTTACTGGCCACATC were designed using Primer Express 3.0 software (Applied Biosystems, Foster city, CA), to yield an amplicon of ∼100 base-pair length. PCR amplification efficiencies were assessed for each primer-set using cDNA equivalents of 1, 2, 5, 10, 20, 50 and 100 ng of total RNA. qRT-PCR was conducted on an ABI Step-One-Plus real-time PCR system (Applied Biosystems, Foster City, CA) in 20 µl reactions. Each reaction consisted of a cDNA equivalent of 20 ng of total RNA, 10 µl Power® SYBR Green PCR Master Mix (Applied Biosystems, Foster City, CA) and forward and reverse primers at 0.9 µM concentration. Each sample was assayed in triplicate for both target and endogenous control, and relative quantitative gene expression was assessed using the ΔΔCT method. The levels of target gene expression were calculated following normalization of endogenous control for each sample, and presented as relative units. The relative gene expression of each tumor was computed by normalizing its value to the tumor with the highest value (a value of 1 corresponds to the tumor with the highest gene expression).

### Histological analysis

For a qualitative histological validation of the imaging and qRT-PCR studies, a separate group of animals (group C, n = 2) was injected at day 7 with the probe at a dose of 455 mg/kg b.w. iodine tagged with rhodamine. At 48-h post-injection, the animals were perfused transcardially with phosphate buffered saline (PBS) followed by 4% paraformaldehyde in PBS. The tumors were explanted and post-fixed overnight in 4% paraformaldehyde in PBS. The fixed tumors were soaked in 30% sucrose (w/v) in PBS at 4°C for cryosectioning. Serial sections of 16 µm thickness were collected using a cryostat (Leica CM 300, Leica, Bannockburn, IL). The tissue slices were immunohistochemically stained for VEGFR-2 using a mouse monoclonal antibody (Santa Cruz Biotechnology, Santa Cruz, CA). The tissues were also stained with the nuclear stain DAPI. The staining procedures followed established methods [34]. The tumor sections were imaged at 4× on the Nikon Eclipse 80i upright microscope using a Microfire CCD camera (Optronics, Golate, CA) that interfaced with the Neurolucida software (MicroBrightField Bioscience, Williston, VT) to obtain a montage of each section. The histological analysis was performed to verify the presence of extravascular intratumoral accumulation of the probe and its location with respect to the tumor vasculature and VEGFR-2 expression.

### Image analysis

The sequential image acquisitions at different time points provided the dynamics of the nanoprobe's accumulation in the tumor over time. The images were analyzed using ImageJ software (NIH, Bethesda, MD) following previously described methods [30], [31].

### Data and statistical analysis

To determine the significance of the relative gene expression levels of the tumors, one-way ANOVA with post-hoc Bonferroni test was performed (SPSS 15, Chicago, IL). A p-value less than 0.05 was used to confirm significant differences at the 95% confidence level. The Anderson-Darling test was performed to verify that the data followed a normal distribution. The tumor signal enhancement profiles and tumor growth curves were fitted into an exponential function [35] using nonlinear regression (Levenberg-Marquardt algorithm) to compute the enhancement rate constant (K^enhancement^) and the tumor growth rate constant (K^tumor growth^), respectively. The K^enhancement^ and K^tumor growth^ constants represent a measure of each animal's tumor vascular permeability to the probe and its tumor growth, respectively. The correlation between the signal enhancement and the tumor growth rate or relative gene expression was determined using Pearson's correlation. A p-value less than 0.05 was used to confirm significant differences at the 95% confidence level. The correctness of the model was determined by examining the residuals plots and other statistical tests.

## Results

### Tumor imaging

**Figure 1:**
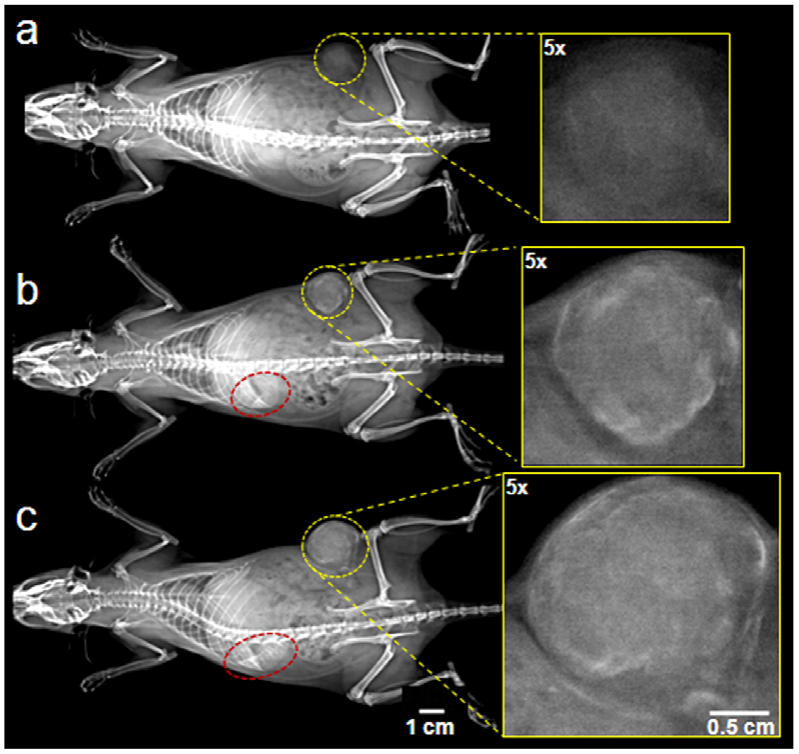
Radiographic images of a rat breast tumor model obtained using a clinical digital mammography system. The images display the 3-day intratumoral fate of the probe (a) before, (b) 24 and (c) 72 h after administration of the probe at a dose of 455 mg/kg b.w. iodine. In the post-injection images no blood vessels were visible in the normal tissue while spleen and tumor were clearly seen. Yellow and brown dotted circles indicate the location of the tumor and spleen, respectively. In the insets, a magnification (5×) of the tumor at each time point is shown.

To eliminate signal from the blood vessels and transparently image the EPR of the probe, contrast-enhanced imaging was performed with IV injection of the probe at a dose of 455 mg/kg b.w. iodine which resulted in a concentration below the threshold for detection of iodine in the blood [31]. This allowed detection of the intratumoral extravascular deposition of the probe with no interference from vascular signal. Fig. 1 shows the pre-injection (t = 0) and post-injection images (t = 24 and 72 h). In the post-injection images, no blood vessels were visible in the normal tissue while the spleen and the tumor were enhanced. Spleen enhancement is consistent with clearance of the liposomal probe via the Reticulo Endothelial System (RES) [36].

### Correlation of angiogenesis biomarkers to EPR imaging

**Figure 2:**
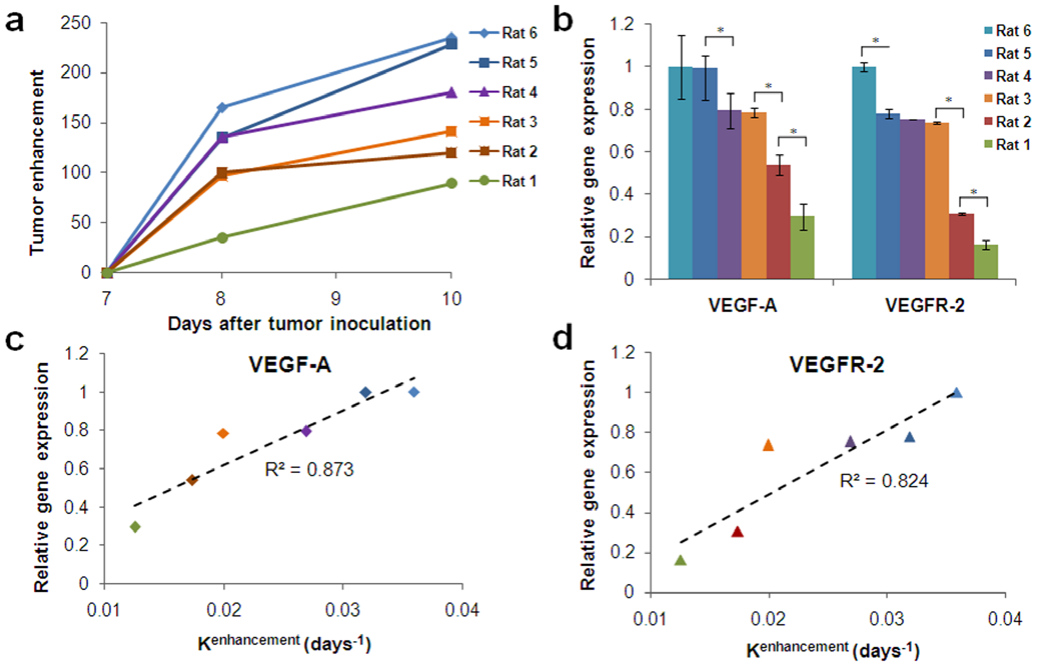
Comparison of the imaging-based EPR measurements to gene expression of VEGF and VEGFR-2. (a) The 3-day pattern of the enhancement upon injection of the probe to a group of rats (n = 6) indicated a high variability in the tumors leakiness. (b) Relative gene expression of VEGF and VEGFR-2 as measured using qRT-PCR also exhibited a high variability (* indicates p<0.005; data presented as mean±standard deviation). (c–d) The relative gene expression level of each tumor was strongly correlated to its tumor enhancement as imaged by mammography (VEGF-A∶ R^2^ = 0.873, p<0.005; VEGFR-2∶ R^2^ = 0.824, p<0.002). Each animal is assigned the same color in all the graphs (e.g. purple indicates animal number 4).

When tumors were monitored for 3 days post-injection, it was observed that the x-ray absorption in tumors due to extravascular nanoprobe varied widely both spatially and temporally suggesting that each tumor had different tumor vessel leakiness. Fig. 2.a summarizes the 3-day time course of the tumor enhancement of a group of animals injected with the nanoprobe (group A). Following imaging, the animals were euthanized, tumors were excised, and total RNA was extracted for qRT-PCR. A significant difference in the gene levels can be observed from one animal to the next (Fig. 2.b). Some tumors displayed 3–5 times less VEGF and VEGFR-2 than others. Importantly, the relative gene expression level of each tumor strongly correlated to its tumor enhancement as imaged by mammography (Fig. 2.c and d).

### Histological evaluation of intratumoral distribution of the probe

**Figure 3:**
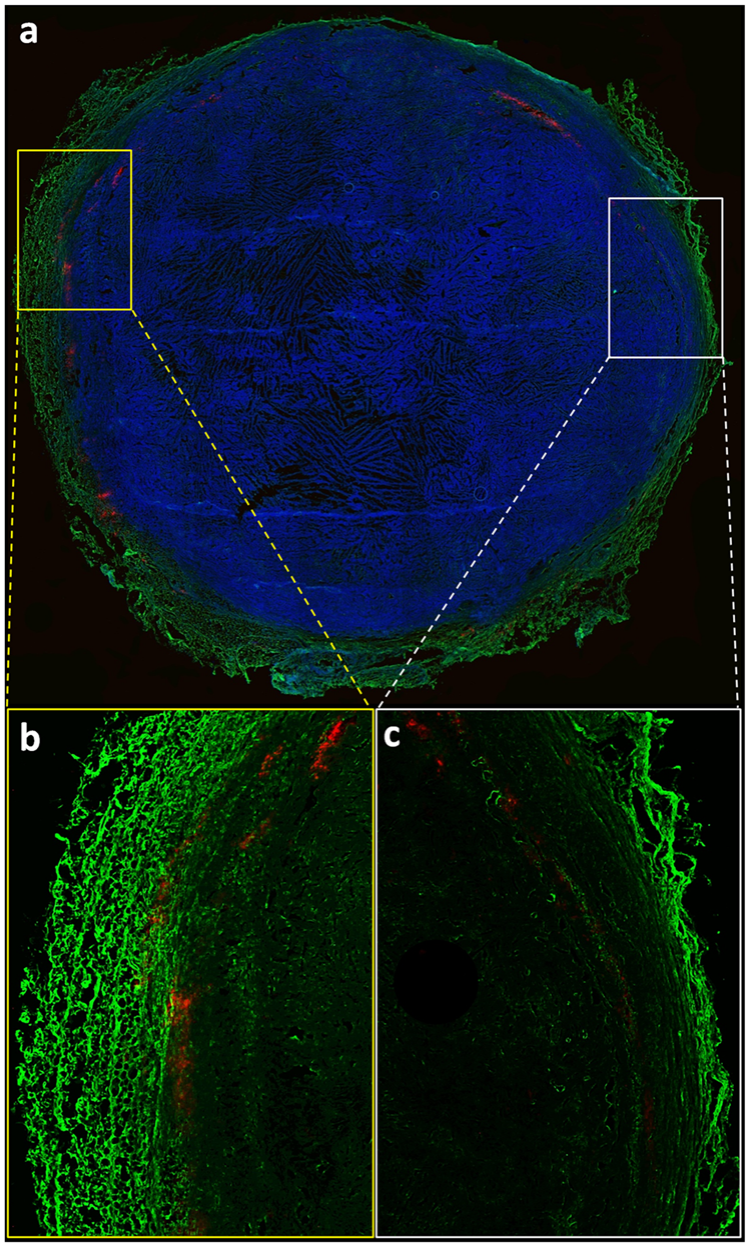
Histological evaluation of the intratumoral microdistribution of the probe and VEGFR-2. (a) The nanoprobes (shown in red) localized in the periphery of the tumor showing a patchy distribution (DAPI was used as a nuclear stain; shown in blue; 4× magnification). In the same slide, the highly vascularized peripheral rim is shown to have high levels of VEGFR-2 (shown in green) compared to the less vascularized inner core. (b) High intratumoral deposition of the nanoprobe is found in a region with high levels of VEGFR-2. (c) Relatively few nanoprobes accumulated in a region of low VEGFR-2 levels.

In a previous study [31], we showed the MAT B III tumor is characterized by a highly vascularized peripheral rim and an internal core with low vascularization. Notably, the extravasated nanoprobes were localized in the well-vascularized periphery of the tumor in a patchy distribution. In the current study, immunofluorescence microscopy was performed to qualitatively determine the microdistribution of VEGFR-2 and nanoprobe deposition. As shown in Fig. 3.a, VEGFR-2 (shown in green) is predominantly found in the periphery whereas lower levels are seen in the inner core of the tumor. The nanoprobe (shown in red) localized in the periphery of the tumor showing a patchy distribution similar to our previous observations [31]. Fig. 3.b and c show two locations from the same histological slide representing two regions with different degree of angiogenesis as indicated by the different levels of VEGFR-2. Of note, more nanoprobes deposited in the region of high levels (Fig. 3.b) than low levels of VEGFR-2 (Fig. 3.c). The deposition of the nanoprobes usually coincided with the regions of high levels of VEGFR-2 which indicates leakier blood vessels. Thus, these data corroborate the imaging data and are consistent with enhanced accumulation of nanoprobes in regions of high angiogenic activity.

### Correlation of tumor growth to imaging of EPR

**Figure 4:**
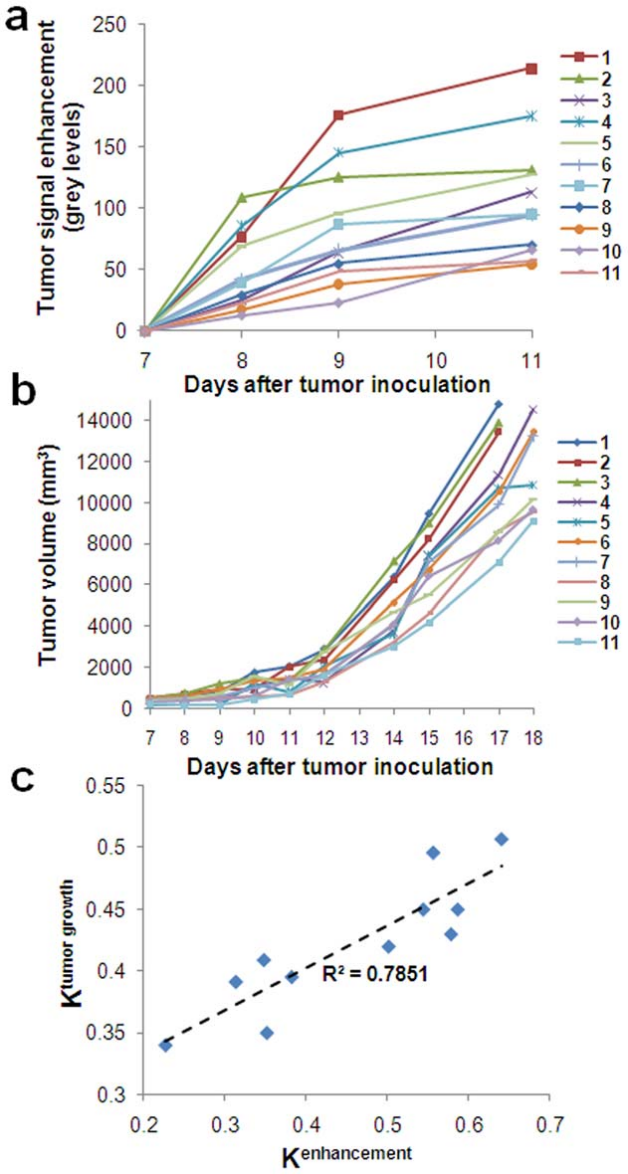
Comparison of the imaging-based measurement of tumor EPR to tumor growth rate. (a) The 4-day pattern of the enhancement following injection of the probe (455 mg/kg iodine) to a group of rats (n = 11) indicated a high variability in the tumors leakiness. (b) The tumor of each animal displayed a different growth rate as indicated by the variable tumor growth curves. (c) The correlation of the tumor growth (K^tumor growth^) and the imaging measurement (K^enhancement^) was statistically significant (R^2^ = 0.785; p<0.001).

To evaluate whether the degree of EPR scales to the tumor growth rate, a different group of animals (group B) was imaged before and after IV injection of the probe and the tumor growth of each animal was measured for several days after imaging. Similarly to before, the tumor enhancement profiles exhibited dissimilar patterns in different animals (Fig. 4.a). Fig. 4.b shows the tumor growth rate of each individual animal displaying a wide variability. Importantly, a significant correlation between the imaging measurements and the tumor growth was observed. Higher intratumoral deposition of the nanocarrier as imaged with mammography indicating leakier vasculature correlated to a faster tumor growth rate. To quantitatively understand the relation of the imaging measurement and tumor growth, we calculated the tumor growth rate constant (K^tumor growth^) and signal enhancement rate constant (K^enhancement^) of each animal. Fig. 4.c demonstrates a strong correlation between K^tumor growth^ and K^enhancement^ with the more leaky tumors (high K^enhancement^) having faster tumor progress (high K^tumor growth^) and vice versa.

## Discussion

Clinical studies have shown the VEGF expression varies among tumors [14]–[16]. In this study, we also observed a high variability in the expression of tumor VEGF-A and VEGFR-2 among the different animals (as measured by qRT-PCR) with the standard deviation being 37 and 52% of the mean value, respectively. This is consistent with a previous study that reported similar variability of angiogenesis biomarkers in animal tumor models [37]. The imaging data verified our hypothesis that the higher expression of angiogenic biomarkers in specific tumors resulted in more permeable blood vessels which subsequently resulted in greater intratumoral accumulation of the nanoprobe. Not surprisingly, we observed that the tumor EPR (as measured by mammography) varied widely from one animal to the next which is consistent with previous studies demonstrating a wide variation in the intratumoral accumulation of nanoscale liposomal agents within tumors of the same ‘stage’ and size in preclinical [38]–[42] and clinical studies [43], [44]. Actually, it is well established that the degree of tumor vasculature leakiness differs not only among same type tumors but even spatially within the same tumor [45]–[47].

Furthermore, the tumors with the leakier blood vessels were the ones with the faster growth rates. Even though the tumor model was developed in a controlled manner by inoculating the same type and number of cells into the same location of rats with the same age and weight, a wide range of tumor growth rates was observed (the standard deviation was about 30% of the mean value). The variable tumor growth rate observed in our study is consistent with human breast tumor xenografts in nude mice where the tumor growth curve had standard deviations of about 15–25% of the mean value [39]. Our results display one more example that tumors represent a very heterogeneous population of different cells with a complex microenvironment that is strongly affected by angiogenesis. Notably, many clinical studies have shown that increased VEGF overexpression in breast tumors correlate with more unfavorable prognosis [14]–[17]. In another clinical study [48], analysis of core biopsies from 155 breast cancer patients showed that tumor angiogenesis correlated with tumor histological analysis suggesting that assessment of tumor angiogenesis can be used as a selection criterion for patients to undergo a more aggressive therapeutic protocol.

The feasibility of our imaging method to evaluate tumor angiogenesis was demonstrated on a single tumor model although human cancer as a disease is much more heterogeneous than one experimental tumor model in terms of both tumors and hosts [49]. The MAT BIII tumor and its vasculature grow rapidly while human tumors exhibit a range of growth rates. To address this variability in tumor growth rates and its relationship to their EPR and angiogenesis status, further testing in more tumor models is required to capture the proliferative range of human tumors.

The significance of tumor vascular permeability and its association to angiogenesis, tumor growth, metastatic tendency, and delivery of macromolecular and nanoscale therapeutics has been well documented and debated [50]. We have recently shown that tumor vascular permeability governs the access of therapeutic agents to tumors [30], [31]. The current study indicates that vessel leakiness is strongly associated with the environment of tumors, the tumor growth and the rate of angiogenesis. Such non-invasive, imaging method can potentially provide an *a priori* determination of the degree of tumor aggressiveness and facilitate personalized therapy. In addition, the realization of the effects of tumor angiogenesis on tumor growth and metastasis [3] has led to the development of anti-angiogenic therapeutic strategies for the treatment of malignant tumors by targeting VEGF signaling [2]. Besides tumor staging and size, the clinician typically has little information to design, track and customize the anti-angiogenic therapy for each tumor in a patient-specific manner [20], [51]. To date, there exist no clinical tools to determine the tumor VEGF expression profile enabling identification of the patients who can possibly benefit from such therapies. In addition, the ability to assess VEGF target inhibition independently of tumor response is critical for these agents, since long times are usually required for changes in tumor growth to become apparent. One recent example demonstrating the critical role that tumor vasculature plays in determining outcomes of antiangiogenic therapies comes from the work of Jain [52], [53], where it is demonstrated that the restructuring of tumor vasculature (a process termed ‘normalization’) leads to better chemotherapeutic outcomes. While antiangiogenic agents focus on destroying tumor related blood vessels compromising the efficiency of subsequent chemotherapy, optimal scheduling and dosing of these therapies can ‘normalize’ the abnormal tumor vasculature for better delivery of oxygen (eliminating hypoxia and its complications) and drugs. Measurement of vessel leakiness using our nanoprobe and mammography can potentially provide prognostic assessment and monitoring of anti-angiogenic therapies of breast cancer. We hypothesize such strategy would also be possible with tomographic methods (e.g. CT) extending the application to other types of cancer.
